# Bilateral Simultaneous Cochlear Implants: How Early Activation Influences Wearing Hours

**DOI:** 10.3390/jcm14030961

**Published:** 2025-02-03

**Authors:** Qusai Tawakkul, Fida Almuhawas, Sarah Alarifi, Nawaf Fatani, Abdulrahman Hagr

**Affiliations:** 1 Department of Otolaryngology-Head and Neck Surgery, College of Medicine, King Saud University, Riyadh 12211, Saudi Arabia; 2 King Abdullah Ear Specialist Center (KAESC), King Saud University Medical City, Riyadh 12372, Saudi Arabia; 3 Research Department, MED-EL Gmbh, Riyadh 11563, Saudi Arabia; ssalarifi@hotmail.com

**Keywords:** data logging, wearing hours, bilateral simultaneous cochlear implant, early activation, cochlear implants

## Abstract

**Background/Objectives**: Cochlear implantation (CI) is a transformative intervention for individuals with sensorineural hearing loss, providing auditory and speech perception improvements. Traditional CI activation occurs 4–6 weeks post-surgery; however, recent advancements allow for early activation within 1–2 days. The integration of data logging in modern CI systems offers objective insights into processor usage and auditory exposure, which are crucial for optimizing rehabilitation outcomes. **Methods**: A retrospective study was conducted on 63 patients with bilateral simultaneous CIs using MED-EL SONNET2/RONDO3 devices. Patients were classified into early activation (*n* = 30, activation within 1–2 days) and classical activation groups (*n* = 33, activation after day 2). Data logging metrics, patient demographics, and implant details were analyzed using the Wilcoxon rank-sum test and Poisson regression. **Results**: Daily processor usage did not significantly differ between groups (9.5 ± 3.0 h/day for classical vs. 9.4 ± 3.7 h/day for early activation, *p* = 0.927). Subgroup analysis showed a significant 18% increase in processor usage with each additional year of patient age (IRR = 1.18, *p* < 0.001) and a 15% decrease in usage with each year delay in implantation age (IRR = 0.85, *p* < 0.001) among early activation users. Switch-on frequencies were comparable between groups, with no significant differences observed (*p* = 1.0). **Conclusions**: Early activation is feasible and associated with consistent CI usage, providing potential benefits in auditory rehabilitation. Future research should explore its impact on long-term speech and language outcomes to inform evidence-based practices.

## 1. Introduction

A cochlear implant (CI) is a surgically implanted neuro-prosthetic used to restore hearing in individuals with sensorineural hearing loss. It is designed to transform acoustic information into an electrical signal that directly stimulates the auditory nerve, particularly the surviving spiral ganglion cell bodies [1]. Using a CI leads to a notable enhancement in auditory and speech perception, mitigating the impact of hearing loss. Traditionally, CI activation requires 4–6 weeks after implantation. Yet, recent progress has substantially shortened this activation period. Some reports [2] indicate that the duration has been dramatically reduced to just one day or even immediately after the surgery, presenting a compelling case for expedited implementation and improved outcomes.

The availability of data regarding the natural auditory settings experienced by individuals with CI has been limited in the past. Previous insights into the usage of processors by CI patients were primarily gathered through patient surveys, which had limitations in capturing a comprehensive and reliable understanding of their daily listening environment [3]. However, to ensure effective hearing rehabilitation and post-operative audiology assessments, it is crucial to have clear and accurate information about an individual’s auditory exposure.

Challenges have been encountered in identifying and evaluating auditory exposure post-implantation. While the correlation between exposure and consistent device usage has been recognized, objective quantification was not feasible until the introduction of the data logging feature in 2013 [3,4]. These technological advancements have provided more accurate methods of assessing and quantifying auditory exposure, facilitating a deeper understanding of the impact of the auditory environment on CI recipients [3]. Data logging has become an integral feature of modern CI systems, providing valuable insights into the usage patterns and listening environments of implant recipients [5]. When integrated into the CI processor, data logging records usage statistics that can be analyzed using clinical programming tools. These data are collected during daily wearing hours, fitting sessions, and routine audiological examinations, allowing for the calculation of average durations spent in different situations. The collected data encompass various parameters, including volume levels, listening conditions, program usage, sound pressure levels (SPLs), microphone sensitivity, directionality, and volume. Additionally, the data log separately documents input from external devices like frequency modulation devices [3]. This comprehensive data collection provides a deeper understanding of the user’s auditory experiences and the factors that influence their hearing outcomes.

Previous studies reported a significant correlation between the average daily utilization of processors and auditory outcomes in children with cochlear implants [6,7,8]. Studies have shown that utilizing a cochlear implant for an average of 12 h per day increases the likelihood of achieving speech recognition benchmarks within one year of activation, as evidenced by sensitivity–specificity curves. In one study, they found that early activation of the cochlear implant within 10 days after surgery is linked to higher device utilization in the early stages and improved speech recognition during both early and late follow-up appointments [9]. These findings highlight the importance of the consistent and prolonged use of CI for optimal hearing outcomes.

Previous research has identified several factors that have been linked to lower device usage among CI users. These factors include younger age, disability, lower maternal education, younger age at cochlear implantation, Medicaid utilization, and a narrower dynamic range [10]. These findings emphasize the importance of understanding the complex interplay of factors that influence CI usage and outcomes.

Despite the importance of data logging analysis in assessing CI effectiveness, and although the early activation of CI has been proposed for more than 10 years [11], no study has yet compared the utilization of data logging in early versus traditionally activated CI users. The standard protocol for adult cochlear implantations is using unilateral or sequential bilateral cochlear implants [12]. However, it was observed in our center that delays in providing patients with bilateral cochlear implantations upfront improves patient outcomes, and it became the standard of care in our center [13].

Therefore, the primary objective of this study was to compare data logging analysis and the rate of processor usage between early and traditionally activated CI users. By examining the data logging metrics of these two approaches, we aim to provide valuable insights into the efficacy and acceptance of early activation and its potential benefits. This comparative analysis could contribute to the ongoing development of evidence-based practices in CI fitting, ultimately improving auditory outcomes and enhancing the quality of life for individuals with hearing impairments.

## 2. Materials and Methods

This retrospective study was conducted at a tertiary university hospital and included patients who underwent cochlear implantation using SONNET2/RONDO3 devices (MED-EL, Innsbruck, Austria). Eligible participants were those with at least one year of CI experience whose data logging metrics were successfully synchronized with the MAESTRO 9.0 fitting software (MED-EL, Innsbruck, Austria) between January 2020 and December 2022. Patients were classified into two groups based on their mode of activation: the early activation group, defined as “patients activated within 1–2 days after surgery” and the classically activated group, defined as “patients activated 4–6 weeks after surgery”. A total of 63 bilateral simultaneous patients were included, with 33 patients in the classical activation group and 30 patients in the early activation group.

Data logging metrics, including the average daily processor usage (hours/day) and the number of times the processor was switched on each day, were extracted manually from the most recent data logs available at the time of the study. Data logs were automatically stored in MAESTRO 9.0 after each programming session, ensuring consistent data collection for all participants. Ethical approval for the study was granted by the Institutional Review Board at King Saud University (E-24-8459) (22 January 2024).

Patients’ demographics as well as data logging information were collected for both groups: the early activation group and the classical activated one. Analysis was conducted using R software version 4.2.2., where descriptive analysis was carried out for quantitative data using mean, standard deviation, and range. For qualitative categorical variables, count and percentage were applied. Comparative analysis between the two activation groups was applied regarding demographics, patients’ characteristics, rate of processor usage (hours/day), and number of switch-on times/day.

For normally distributed continuous data, the independent samples *t*-test was used for comparative analysis, while the Wilcoxon rank-sum test was used when data violated the normality assumptions, and the chi-squared test or Fisher’s exact test was used for categorical data. The Poisson regression model was applied to investigate the potential predictors for the rate of processor usage in hours/day, while the ordinal logistic regression model was applied to predict the number of switch-on times per day, categorized as being from 1 to 5, from 5 to 10, and >10 times/day. Normality was checked using the Shapiro–Wilk test, and *p* values ≤ 0.05 were considered statistically significant.

## 3. Results

### 3.1. Baseline Demographics and Patient Data Logging Characteristics in Table 1

This study included 63 patients divided into two groups: the classical activation group (*n* = 33) and the early activation group (*n* = 30). The gender distribution was comparable between the two groups, with females comprising 57.6% of the classical activation group and 46.7% of the early activation group, while males accounted for 42.4% and 53.3%, respectively. This difference was not statistically significant (*p* = 0.54). The mean age of participants was slightly higher in the classical activation group (9.2 ± 9.4 years) compared to the early activation group (8.6 ± 9.9 years). This difference was statistically significant (*p* = 0.014). The age range spanned from 5.1 to 44.8 years in the classical activation group and 3.5 to 42.8 years in the early activation group. The mean age at implantation was 5.2 ± 9.2 years in the classical activation group and 5.6 ± 9.7 years in the early activation group, showing a significant difference between the groups (*p* = 0.043). The range of ages at implantation was 2.2 to 40.6 years for the classical activation group and 1.2 to 39.3 years for the early activation group.

Participants in the classical activation group had a significantly longer mean duration since implantation (3.9 ± 1.8 years) compared to those in the early activation group (3.0 ± 1.4 years, *p* < 0.001). The duration ranged from 2.4 to 9.9 years in the classical activation group and 1.5 to 7.8 years in the early activation group. All patients (100%) in both groups underwent bilateral simultaneous cochlear implantation, with no differences observed between the groups (*p* = 1.0). The distribution of left ear implants was 48.5% in the classical activation group and 46.7% in the early activation group. Right ear implants accounted for 51.5% and 53.3%, respectively. No significant differences were noted between the groups (*p* = 1.0). The mean duration of follow-up was 75.1 ± 47.6 days in the classical activation group and 89.8 ± 113.1 days in the early activation group. The difference in follow-up duration was not statistically significant (*p* = 0.454).

**Table 1:** Comparative analysis for baseline demographics, patients’ characteristics, and data logging information between classical and early activation groups.

Baseline Demographics and Patients’ Characteristics		Classical Activation (*n* = 33)	Early Activation (*n* = 30)	Total (*n* = 63)	*p* Value
Gender	Female	19 (57.6)	14 (46.7)	33 (52.4)	0.54
	Male	14 (42.4)	16 (53.3)	30 (47.6)	
Age (Years)	Mean (SD)	9.2 (9.4)	8.6 (9.9)	8.9 (9.6)	0.014
	Min–Max	5.1–44.8	3.5–42.8	3.5–44.8	
Age at Implantation (Years)	Mean (SD)	5.2 (9.2)	5.6 (9.7)	5.4 (9.3)	0.043
	Min–Max	2.2–40.6	1.2–39.3	1.2–40.6	
CI Duration (Years)	Mean (SD)	3.9 (1.8)	3.0 (1.4)	3.5 (1.7)	<0.001
	Min–Max	2.4–9.9	1.5–7.8	1.5–9.9	
Mode of Intervention	Bilateral Simultaneous	33 (100.0)	30 (100.0)	63 (100.0)	1.0
Ear	Left	16 (48.5)	14 (46.7)	30 (47.6)	1.0
	Right	17 (51.5)	16 (53.3)	33 (52.4)	
Coil Type	D Coil	2 (6.1)	2 (6.7)	4 (6.3)	1.0
	DL Coil	31 (93.9)	28 (93.3)	59 (93.7)	
Duration (Days)	Mean (SD)	75.1 (47.6)	89.8 (113.1)	82.0 (84.3)	0.454
	Min–Max	21.0–220.0	24.0–611.0	21.0–611.0	
Data Logging					
Processor Usage (Hours/Day)	Mean (SD)	9.5 (3.0)	9.4 (3.7)	9.5 (3.3)	0.927
	Min–Max	4.1–13.7	1.2–20.5	1.2–20.5	
Switch-On Times/Day	from 1 to 5	23 (69.7)	21 (72.4)	44 (71.0)	1.0
	from 5 to 10	9 (27.3)	7 (24.1)	16 (25.8)	
	>10	1 (3.0)	1 (3.4)	2 (3.2)	

Data are represented as mean (standard deviation) and range or count (percentage).

### 3.2. Predicting the Incidence Rate Ratio (IRR) of Processor Usage Hours per Day

**Figure 1:**
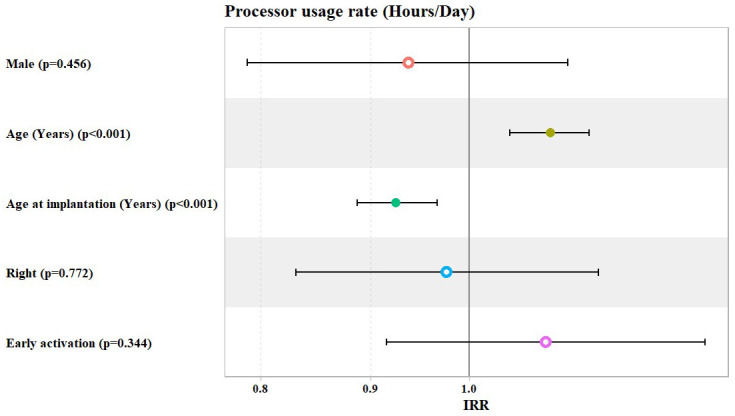
Potential predictors for incidence rate ratio (IRR) of the processor usage hours per day.

The overall analysis showed that, after adjusting for patients’ gender, age, age at implantation, laterality, and mode of activation, the adjusted model showed a statistically significant increase in the IRR of processor usage hours/day of about 9% for each one-year increase in patients’ age (IRR = 1.09, 95% Confidence Interval: (1.04 to 1.14), *p* value < 0.001), while this decreased significantly by about 8% for each one-year increase in patients’ age at implantation (IRR = 0.92, 95% Confidence Interval: (0.89 to 0.97), *p* value < 0.001). However, the mode of activation did not show any significant association with the rate of processor usage hours per day, keeping other demographics (gender, age, age at implantation, and laterality) constant (Table 2 and Figure 1).

#### For Subgroup Analysis

**Figure 2:**
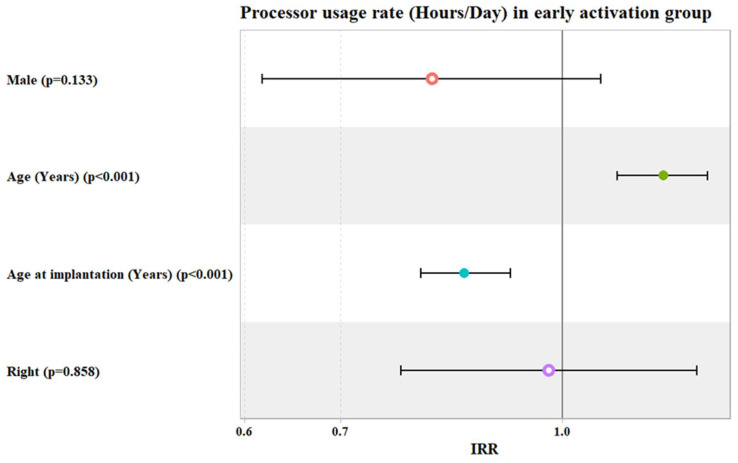
Potential predictors for incidence rate ratio (IRR) of the processor usage hours per day in early activation group.

In the early activation group, after adjusting for patients’ gender, age, age at implantation, and laterality, the adjusted IRR of processor usage hours/day increased significantly by about 18% for each one-year increase in patients’ age (IRR = 1.18, 95% Confidence Interval: (1.09 to 1.26), *p* value < 0.001). Meanwhile, this decreased significantly by about 15% for each one-year increase in patients’ age at implantation (IRR = 0.85, 95% Confidence Interval: (0.80 to 0.92), *p* value < 0.001) (Table 3 and Figure 2).

**Figure 3:**
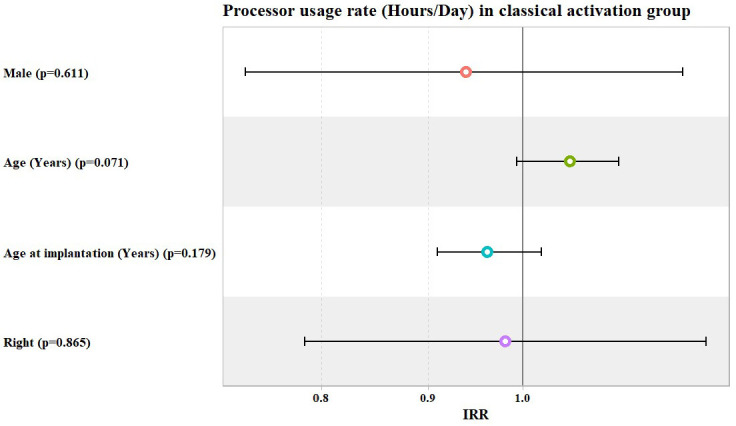
Potential predictors for incidence rate ratio (IRR) of the processor usage hours per day in classical activation group.

In the classical activation group, the IRR of processor usage hours/day in the univariate analysis showed a statistically significant increase by about 1% for each one-year increase in either patients’ age or age at implantation (IRR = 1.01, 95% Confidence Interval: (1.00 to 1.02), *p* value = 0.012) and (IRR = 1.01, 95% Confidence Interval: (1.00 to 1.02), *p* value = 0.027), respectively. However, these values lost their significance after adjusting for other patients’ demographics (gender and laterality) (Table 4 and Figure 3).

### 3.3. Predicting the Odds of Switch-On Times per Day, Categorized as Being from 1 to 5, from 5 to 10, and >10 Times/Day

**Figure 4:**
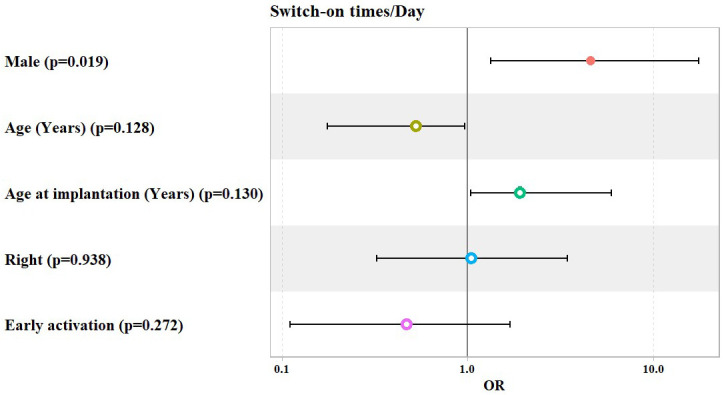
Potential predictors for Odds Ratio (OR) of switch-on times per day.

The overall analysis showed that, after adjusting for patients’ gender, age, age at implantation, laterality, and mode of activation, the adjusted model showed that the odds of having 5 to 10 or more than 10 switch-on times per day (versus 1 to 5 times per day) increased significantly by about 4.6 fold among males compared to female patients (OR = 4.58, 95% Confidence Interval: (1.33–17.54), *p* value = 0.019), keeping other patients’ demographics (age, age at implantation, laterality, and mode of activation) constant. Meanwhile, the mode of activation did not show any significant association with switch-on times per day (Table 5 and Figure 4).

#### For Subgroup Analysis

**Figure 5:**
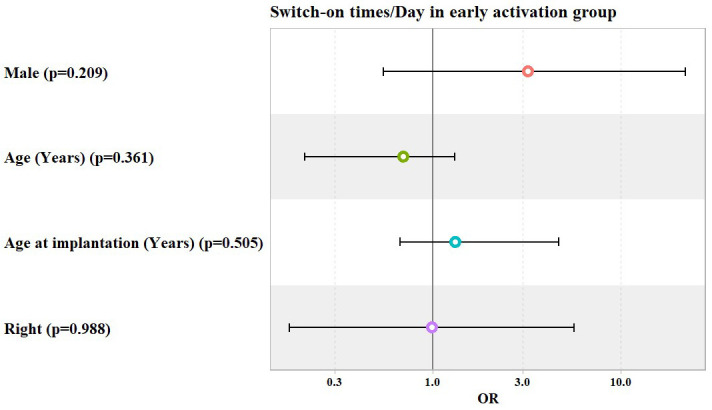
Potential predictors for Odds Ratio (OR) of switch-on times per day in early activation group.

**Figure 6:**
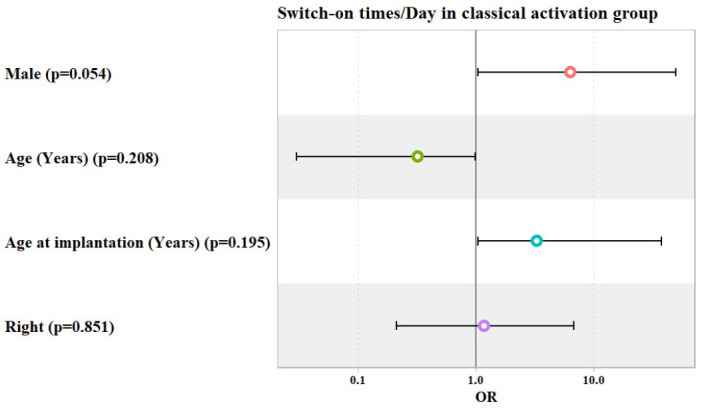
Potential predictors for Odds Ratio (OR) of switch-on times per day in classical activation group.

In both the early and classical activation groups, patients’ gender, age, age at implantation, and laterality did not show any significant association with switch-on times per day, as shown in Table 6 and Table 7 and Figure 5 and Figure 6.

## 4. Discussion

This study examined the differences in daily switch-on timings and processor use hours between classical and early bilateral simultaneous cochlear implant fittings. Our results demonstrate that there was no significant difference (*p* = 0.927) in processor utilization (measured in hours per day) between the early fitting and classical activation groups. Thus, the switch-on timings per day between the early and classical fit groups did not statistically significantly differ.

Within the subgroup analysis of the early activation group, there was a significant correlation between patient age and processor usage hours. Specifically, for each additional year of patient age, there was an 18% increase in processor usage (IRR = 1.18, *p* < 0.001). This finding indicates that as children age, their proficiency in using the cochlear implant increases, possibly because of enhanced cognitive and motor abilities that assist in maintaining and the upkeep of the device. Additionally, the correlation between the age at which the implant is placed and the number of hours it is used emphasizes the advantages of fitting the implant at an early stage. More precisely, for every additional year of age at the time of implantation, there was a 15% reduction in consumption. The incidence rate ratio (IRR) was calculated to be 0.85, with a *p* value of less than 0.001.

These findings indicate that implanting the device earlier results in more regular and extended processor usage, possibly because the younger brain has greater neuronal plasticity and adaptability during early development [14]. A study by Gagnon et al. discovered that spoken language abilities at age three were predicted by both age at implantation and cumulative hearing hour percentage (HHP). Although it does not measure wearing hours directly, this points to a possible correlation between age at implantation and device usage [15].

Moreover, another study found that young children with cochlear implants used their devices for an average of 6.7 h per day. They also reported that longer daily device use was significantly correlated with younger age at cochlear implantation and longer device experience [16].

Several studies have highlighted the importance of consistent and prolonged CI use in children.

Alhabib et al. reported a significant positive correlation (r = 0.54, *p* = 0.0009) between daily use of an audio processor and speech discrimination scores in prelingual CI users. A minimum of 8.3 h/day of CI use was needed to achieve acceptable language development [17].

Thus, early fitting not only enhances immediate auditory outcomes but also contributes to sustained and increased usage of the device, promoting better long-term developmental outcomes. Early fitting is safe and feasible for many patients. This approach allows for earlier rehabilitation and may have long-term benefits for stimulation levels and dynamic range [18].

Bilateral simultaneous cochlear implantation (BiCI) is an well-known and effective approach for pediatric, and nowadays, this is an accepted approach for adult patients too, showing safety and efficacy in improving speech outcomes [19,20,21,22]. Much research has confirmed that simultaneous BiCI in adults is safe, with recovery times comparable to those for unilateral implantation and with fewer complications [13,23]. Another study comparing bilateral simultaneous and bilateral sequential cochlear implantation discovered that bilateral simultaneous cochlear implantation helps children with language, selective attention, and coping with the Stroop effect [24]. This approach reduces the number of surgeries and hospitalizations and shortens operating time, facilitating faster binaural hearing restoration. A randomized trial revealed that simultaneous BiCI provides comparable overall hearing benefits to sequential BiCI, with notable improvements in speech intelligibility in noisy environments [25].

Several studies in the literature provide evidence that early activation of cochlear implants is both safe and effective, enabling more efficient auditory and speech rehabilitation. Post-operative activation of cochlear implants was assessed for safety in one study. The study revealed that early activation had no detrimental impact on wound healing or resulted in increased pain [2,11].

A recent study examined the effects of early activation of cochlear implants on electrode impedance in children. The study revealed that the electrode impedance in the group that underwent early activation was significantly lower compared to the control group, up until one month following the surgery. Nevertheless, the electrode impedance at the twelve-month mark following the cochlear implant was comparable in both groups [26].

This study has some limitations that are worth mentioning. It was noted in Table 1 that there were some significant differences between the two groups (classical vs. early). One of these differences was the duration since implantation, where patients who used the classical activation technique had a significantly longer duration. This is explained by the fact that classical activation was the standard approach in our center before transitioning to the early activation method.

The retrospective nature of this study limits its ability to establish causation between activation time and processor usage; therefore, prospective studies should provide stronger evidence about processor wearing hours.

## 5. Conclusions

This study underscores the importance of considering early fitting when evaluating cochlear implant usage. Early activation showed a non-significant difference in the daily wearing hours of the processor from classical activation. This may enhance usage among younger patients due to better adaptation capabilities. This study highlights several areas for future research to enhance understanding of cochlear implant (CI) activation and optimization. Conducting a prospective trial would provide stronger evidence for the observed differences in CI usage and outcomes.

## Figures and Tables

**Table 2:** Univariate and multivariate Poisson regression models used to investigate the potential predictors for the rate of processor usage in hours/day (overall).

Predictors	Crude IRR (Univariate)		*p* Value	Adjusted IRR (Multivariate)		*p* Value
	IRR	95% CI		IRR	95% CI	
Male	1.02	0.87 to 1.20	0.812	0.94	0.79 to 1.11	0.456
Age	1.01	1.00 to 1.02	0.005	1.09	1.04 to 1.14	<0.001
Age at Implantation	1.01	1.00 to 1.02	0.032	0.92	0.89 to 0.97	<0.001
Right Ear	0.97	0.83 to 1.14	0.747	0.98	0.83 to 1.15	0.772
Early Activation	0.99	0.84 to 1.17	0.919	1.09	0.92 to 1.29	0.344

IRR = incidence rate ratio; 95% CI = 95% Confidence Interval.

**Table 3:** Univariate and multivariate Poisson regression models used to investigate the potential predictors for the rate of processor usage in hours/day (in early activation group).

Predictors	Crude IRR (Univariate)		*p* Value	Adjusted IRR (Multivariate)		*p* Value
	IRR	95% CI		IRR	95% CI	
Male	1.03	0.81 to 1.30	0.819	0.81	0.62 to 1.06	0.133
Age	1.01	1.00 to 1.02	0.154	1.18	1.09 to 1.26	<0.001
Age at Implantation	1.00	0.99 to 1.02	0.408	0.85	0.80 to 0.92	<0.001
Right Ear	0.97	0.76 to 1.23	0.78	0.98	0.77 to 1.24	0.858

IRR = incidence rate ratio; 95% CI = 95% Confidence Interval.

**Table 4:** Univariate and multivariate Poisson regression models used to investigate the potential predictors for the rate of processor usage in hours/day (in classical activation group).

Predictors	Crude IRR (Univariate)		*p* Value	Adjusted IRR (Multivariate)		*p* Value
	IRR	95% CI		IRR	95% CI	
Male	1.01	0.81 to 1.27	0.9	0.94	0.74 to 1.19	0.611
Age	1.01	1.00 to 1.02	0.012	1.05	0.99 to 1.11	0.071
Age at Implantation	1.01	1.00 to 1.02	0.027	0.96	0.91 to 1.02	0.179
Right Ear	0.98	0.79 to 1.22	0.857	0.98	0.79 to 1.22	0.865

IRR = incidence rate ratio; 95% CI = 95% Confidence Interval.

**Table 5:** Ordinal logistic regression model used to predict the number of switch-on times per day, categorized as being from 1 to 5, from 5 to 10, and >10 times/day (overall).

Predictors	Odds Ratio (OR)	95% Confidence Interval		*p* Value
		Lower	Upper	
Male	4.58	1.33	17.54	0.019
Age (Years)	0.53	0.17	0.96	0.128
Age at Implantation (Years)	1.92	1.04	5.95	0.130
Right Ear	1.05	0.32	3.42	0.938
Early Activation	0.47	0.11	1.7	0.272

**Table 6:** Ordinal logistic regression model used to predict the number of switch-on times per day, categorized as being from 1 to 5, from 5 to 10, and >10 times/day, in early activation group.

Predictors	Odds Ratio (OR)	95% Confidence Interval		*p* Value
		Lower	Upper	
Male	3.2	0.54	22.05	0.209
Age (Years)	0.7	0.21	1.3	0.361
Age at Implantation (Years)	1.32	0.66	4.64	0.505
Right Ear	0.99	0.17	5.63	0.988

**Table 7:** Ordinal logistic regression model used to predict the number of switch-on times per day, categorized as being from 1 to 5, from 5 to 10, and >10 times/day, in classical activation group.

Predictors	Odds Ratio (OR)	95% Confidence Interval		*p* Value
		Lower	Upper	
Male	6.34	1.05	50.08	0.054
Age (Years)	0.32	0.03	0.99	0.208
Age at Implantation (Years)	3.32	1.05	37.7	0.195
Right Ear	1.18	0.21	6.85	0.851

## Data Availability

Data are contained within the article.
